# Clinical silence of pulmonary lymphoepithelioma-like carcinoma with subcutaneous metastasis: a case report

**DOI:** 10.1186/s12957-019-1671-z

**Published:** 2019-07-24

**Authors:** Takafumi Shima, Kohei Taniguchi, Yasutsugu Kobayashi, Shotaro Kakimoto, Nagahisa Fujio, Kazuhisa Uchiyama

**Affiliations:** 1 Department of General and Gastroenterological Surgery, Osaka Medical College, 2-7 Daigaku-machi, Takatsuki, Osaka, 569-8686 Japan; 2 Translational Research Program, Osaka Medical College, 2-7 Daigaku-machi, Takatsuki, Osaka, 569-8686 Japan; 3 Department of Pathology, Minami Osaka Hospital, 1-18-18 Higashikagaya, Suminoe-ku, Osaka, 559-0012 Japan; 4 Department of Thoracic Surgery, Minami Osaka Hospital, 1-18-18 Higashikagaya, Suminoe-ku, Osaka, 559-0012 Japan; 5 Department of Surgery, Minami Osaka Hospital, 1-18-18 Higashikagaya, Suminoe-ku, Osaka, 559-0012 Japan

**Keywords:** Lymphoepithelioma-like carcinoma, Lung cancer, Cutaneous metastasis, Epstein-Barr virus

## Abstract

**Background:**

Dissemination of lung cancer to cutaneous sites usually results in a poor prognosis. Pulmonary lymphoepithelioma-like carcinoma (PLELC) is a rare tumor, and no therapeutic strategy for it has yet been established. We present herein an extremely rare case of a long-term surviving patient with PLELC showing subcutaneous metastasis.

**Case presentation:**

A 76-year-old woman was diagnosed unexpectedly as having PLELC based on a nodule on her back. After surgical resection of the primary and metastatic lesions, she has remained alive with no recurrence for over 5 years without any additional therapy.

**Conclusion:**

Even in the case of PLELC with subcutaneous metastasis, surgical management may afford a prognosis of long-term survival.

**Electronic supplementary material:**

The online version of this article (10.1186/s12957-019-1671-z) contains supplementary material, which is available to authorized users.

## Background

Lung cancer with cutaneous metastases represents a grave disease with poor prognostic [1]. Pulmonary lymphoepithelioma-like carcinoma (PLELC) is a rare form of cancer that shares morphological similarities to undifferentiated nasopharyngeal carcinoma, and there is no unified therapeutic strategy for it [2, 3]. We herein report an extremely rare case of a long-term surviving patient having clinically silent PLELC with subcutaneous metastasis.

## Case presentation

**Fig. 1:**
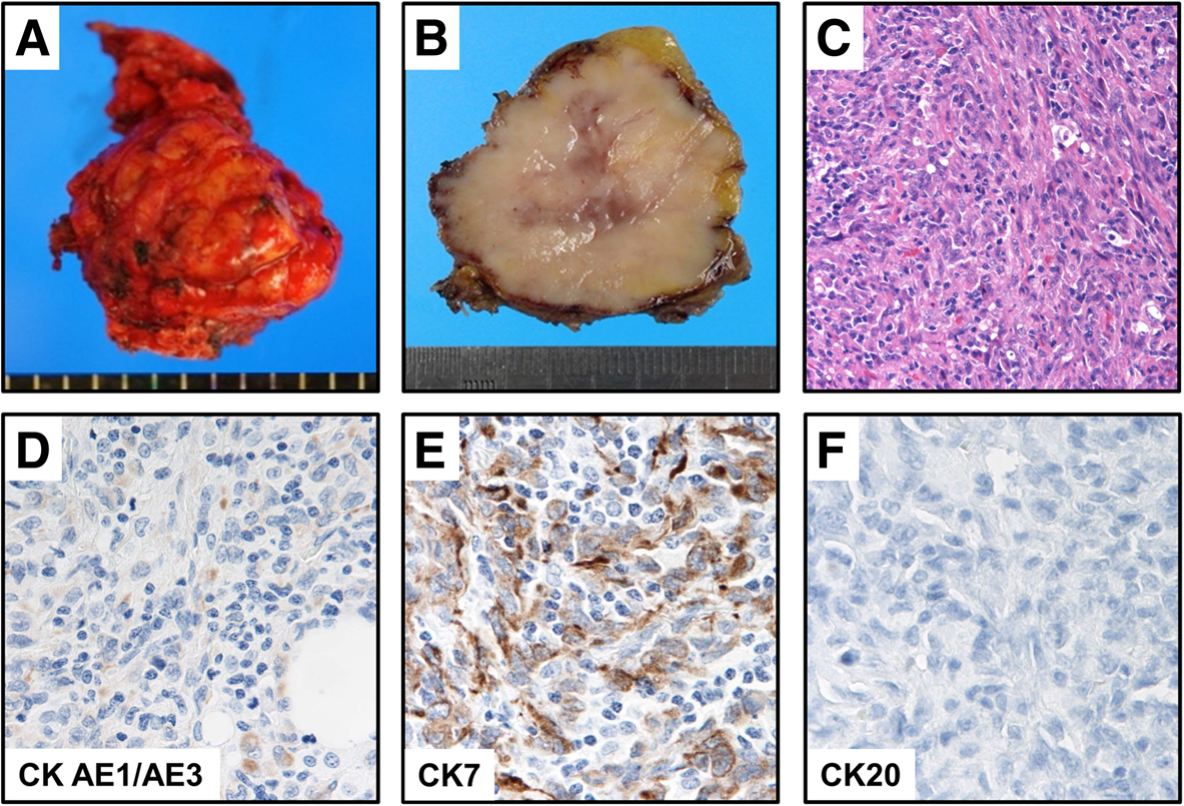
**a** Operative specimen revealed a subcutaneous tumor measuring 40 × 40 mm. **b** Split surface of the tumor. **c** Histopathological examination with hematoxylin and eosin staining of the tumor showed evidence of malignant spindle-cell proliferation (× 200). **d**–**f** Immunohistochemistry showed that these tumor cells were positive for CK AE1/AE3 (**d**; × 400) and CK7 (**e**; × 400), but negative for CK20 (**f**; × 400)

The patient was a 76-year-old Japanese woman with a primary complaint of a subcutaneous nodule in her back. She had a smoking history of 180 pack-years spanning the period from when she was 20 years old through her 73rd year. We suspected the tumor of being a lipoma, and so tumorectomy was performed as usual with no other preoperative inspection. A specimen of the tumor revealed a subcutaneous solid tumor measuring 40 × 40 mm (Fig. 1a, b). Hematoxylin and eosin (HE) staining showed evidence of malignant spindle-cell proliferation (Fig. 1c). Also, immunohistochemistry (IHC) showed that these tumor cells were positive for cytokeratins (CK) AE1/AE3 and CK7 and negative for CK20 (Fig. 1d–f). Also, the cells were negative for all mesenchymal markers tested (data not shown). Therefore, subcutaneous metastasis of an epithelial tumor of unknown origin was suspected.

**Fig. 2:**
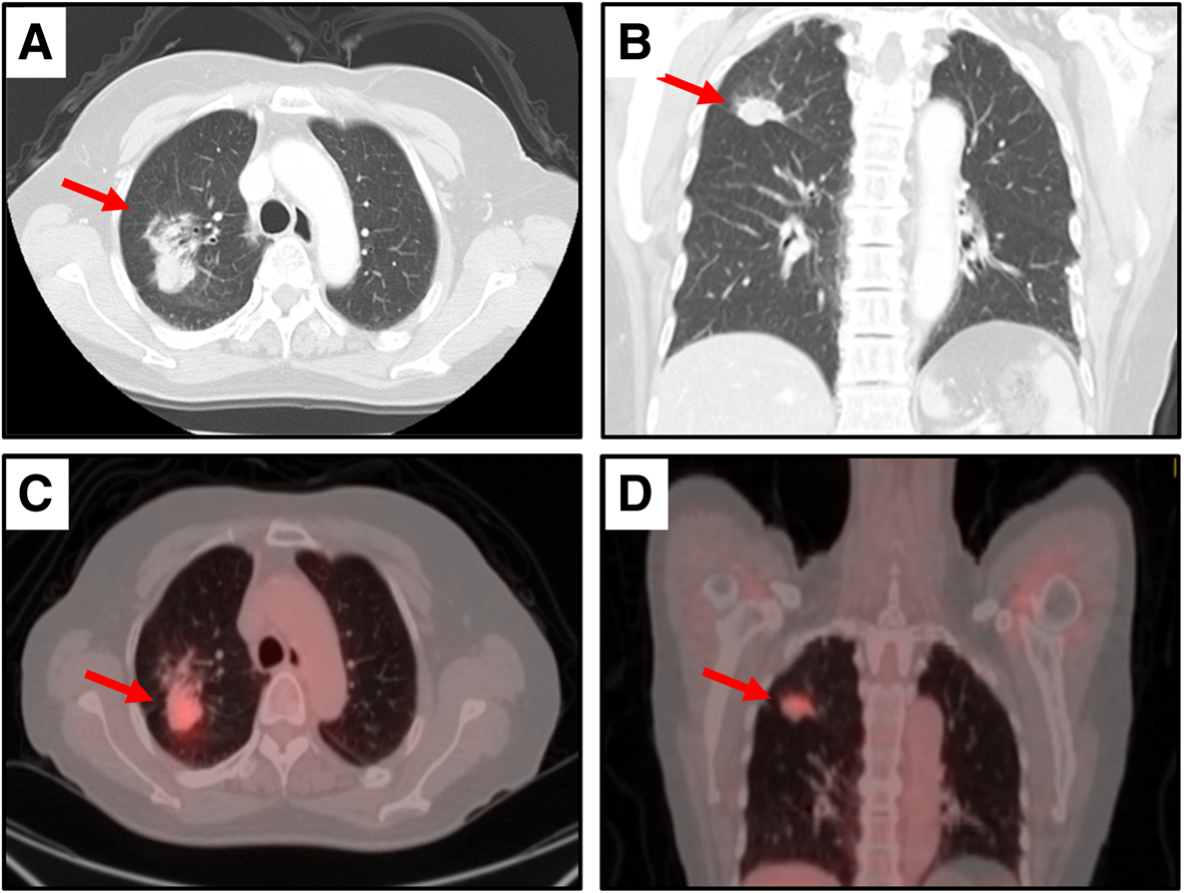
Imaging findings for patient. **a**, **b** Chest computed tomography scan revealed an irregular tumor in the right upper lobe, which tumor was suspected to mean lung cancer (arrow). **c**, **d** Positron emission tomography revealed high levels of accumulation of ^18^F-fludeoxyglucose in the right upper lobe (arrow) (maximum standardized uptake value of 4.09)

**Fig. 3:**
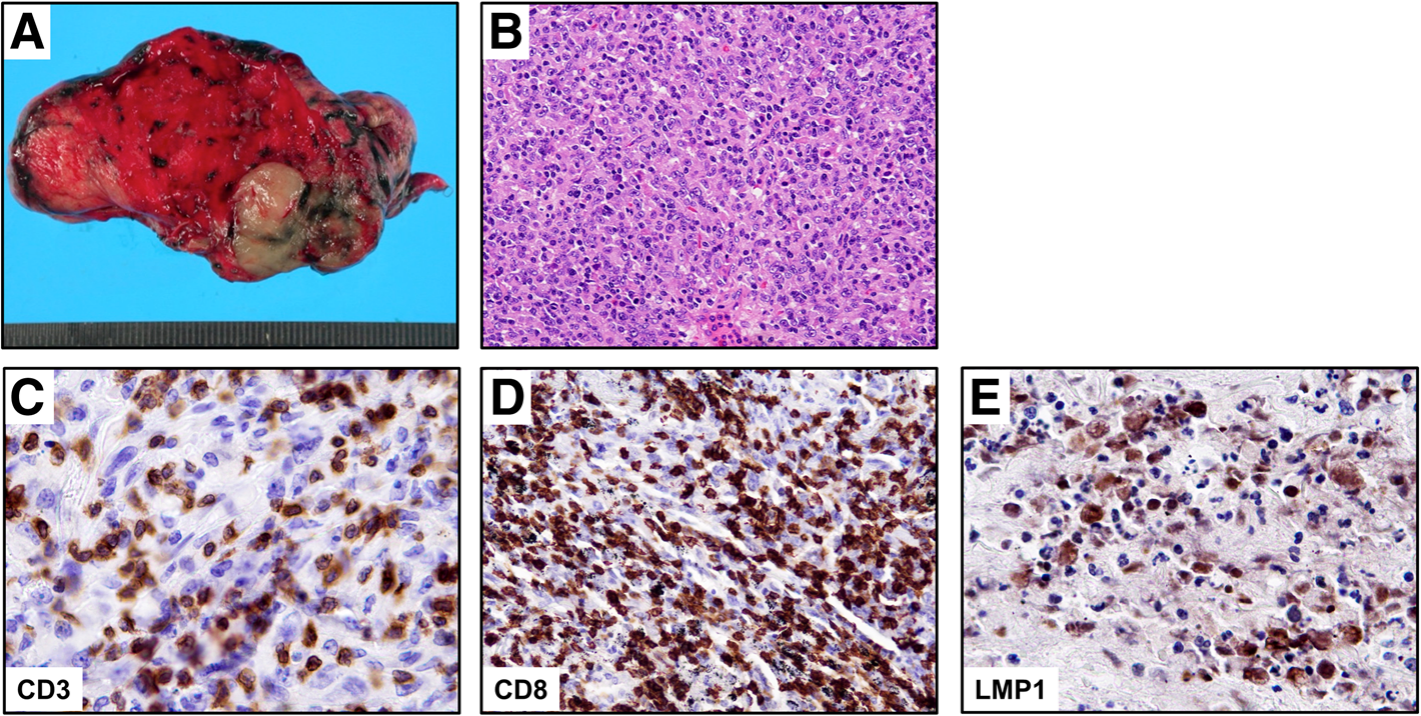
**a** Operative specimen revealing a lung tumor measuring 40 × 30 mm. **b** Histopathological examination with hematoxylin and eosin staining of the tumor showed an undifferentiated carcinoma with poorly defined nests of neoplastic cells and a stroma exhibiting prominent lymphoplasmacytic infiltration (× 200). **c**, **d** Positive staining for CD3 (**c**; × 400) and CD8 (**d**; × 200) in lymphocytes around the tumor. **e** Positive staining for latent membrane protein 1 (× 400)

**Fig. 4:**
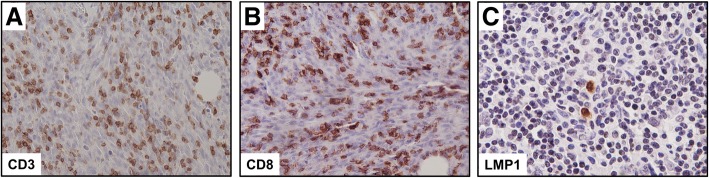
**a**–**c** Immunohistochemistry of the subcutaneous tumor. These tumor cells gave positive staining for CD3 (**a**; × 200), CD8 (**b**; × 200) and latent membrane protein 1 (**c**; × 400)

Her laboratory data including tumor markers showed no abnormalities. Also, bronchofiberscopic washing cytology was negative. However, a chest computed tomography (CT) scan and positron emission tomography (PET) revealed an irregular tumor in the right upper lobe (Fig. 2a–d). Abnormal findings to suggest a nasopharyngeal tumor were not detected by the CT scan or PET (data not shown). Hence, in order to make a definite diagnosis, right upper lobectomy and lymph node dissection were performed. An operative specimen revealed a lung tumor measuring 40 × 30 mm (Fig. 3a). HE staining of the tumor showed an undifferentiated carcinoma with poorly defined nests of neoplastic cells and a stroma exhibiting prominent lymphoplasmacytic infiltration (Fig. 3b). Also, CD3 and CD8 were detected in the lymphocytes around the tumor (Fig. 3c, d). In situ hybridization for Epstein-Barr virus (EBV)-encoded RNA (EBER) gave a negative result (data not shown), but IHC showed the tumor to be positive for latent membrane protein 1 (LMP1; Fig. 3e). Also, IHC showed that the tumor was negative for p63 and thyroid transcription factor-1 (TTF-1; Additional file 1 a, b). The Ki-67 cell proliferation index was approximately 70–80% (Additional file 1 c). There was no lymph node metastasis. These morphologies were similar to those of her subcutaneous tumor. Indeed, the same results of IHC were obtained for the subcutaneous tumor specimens (Fig. 4a–c). Based on these findings, we concluded the patient to have PLELC with subcutaneous metastasis (pT2a pN0 M1b pStage IVA). Because the patient declined chemotherapy, we observed her carefully without it. The patient was followed every 3 months by performing a blood test that included a tumor marker and by doing a semi-annual CT scan. Over the past 5 years, she has remained alive and with no evidence of recurrence.

## Discussion and conclusions

PLELC is very rare and was first described by Bégin and colleagues [4] in 1987, and it is an EBV-associated epithelial neoplasm. PLELC is generally found in non-smoking, younger patients, and women [2]. The diagnosis of PLELC depends mainly on morphologic characteristics, which includes solid nests of tumor cells with prominent nucleoli in a syncytial arrangement surrounded by lymphocyte infiltrates. Also, focal squamous and spindle cell differentiation can occur [2, 3]. The presence of EBER, LMP1, and CD8-positive T lymphocytes suggest a relationship between EBV and the pathogenesis of the tumor [2]. Among them, EBER and LMP1 are routinely used to detect a latent EBV infection in patients diagnosed with an EBV-associated tumor [5–7]. In this present case, in situ hybridization for EBER gave a negative result, but IHC findings for LMP1 and CD8 were positive. Hence, we considered the tumor to be associated with an EBV infection. An earlier report of an EBER-negative PLELC case, as in the present case, supports our consideration [8]. Although the patient was a smoker and an old woman, we concluded our case to be PLELC in consideration of the pathological findings.

It is known that patients with PLELC have a better prognosis than those having other types of non-small cell lung cancer (NSCLC) [3]. However, the prognosis of stage IV-PLELC is not better than that of NSCLC [9, 10]. As to the treatment for PLELC, complete resection is the primary approach to obtain a cure, but treatment for advanced PLELC is controversial, owing to its being a rare entity [3]. Hence, the accumulation of more cases and sharing of the clinical experience of PLELC should be required.

Sometimes, cutaneous metastases may be the first indication of clinically silent visceral malignancies [11]. Cutaneous metastases represent a grave prognostic sign, particularly in patients with lung cancer [1]. To our knowledge, there are no reports of PLELC with subcutaneous metastasis. Fortunately, the present case showed long-term survival. It is difficult to give a reason for it, but a couple of factors may be an isolated metastasis and pathological type. As in the present case, if a cutaneous metastasis is isolated, resection of the primary and metastatic lesions may afford a prognosis of long-term survival. Especially in the case of PLELC, surgical complete resection should be considered.

In summary, we experienced an extremely rare case of a PLELC patient with a subcutaneous metastasis who showed long-term survival. We propose that prompt diagnosis is indispensable in the case of a subcutaneous lesion and that a whole-body search should be conducted immediately to determine the site of the primary lesion. Also, appropriate surgical resection should be performed to increase the chance of long survival of such patients.

## Additional file

Additional file 1: (A, B) Immunohistochemistry showed that the lung tumor cells were negative for p63 (A; × 200) and thyroid transcription factor-1 (B; × 200). (C) The Ki-67 cell proliferation index was approximately 70–80% (× 200). (TIF 9365 kb)

## Data Availability

Data supporting the conclusions of this study are included in this published article.

## Abbreviations

CK: Cytokeratins
CT: Computed tomography
EBER: Epstein-Barr virus-encoded RNA
EBV: Epstein-Barr virus
HE: Hematoxylin and eosin
IHC: Immunohistochemistry
LMP1: Latent membrane protein 1
NSCLC: Non-small cell lung cancer
PET: Positron-emission tomography
PLELC: Pulmonary lymphoepithelioma-like carcinoma
TTF-1: Thyroid transcription factor-1
