# Supplementary figures and images for: Clinical silence of pulmonary lymphoepithelioma-like carcinoma with subcutaneous metastasis: a case report
Source: World J Surg Oncol. 2019 Jul 24;17:128. doi: 10.1186/s12957-019-1671-z (PMC6652011; doi:10.1186/s12957-019-1671-z)

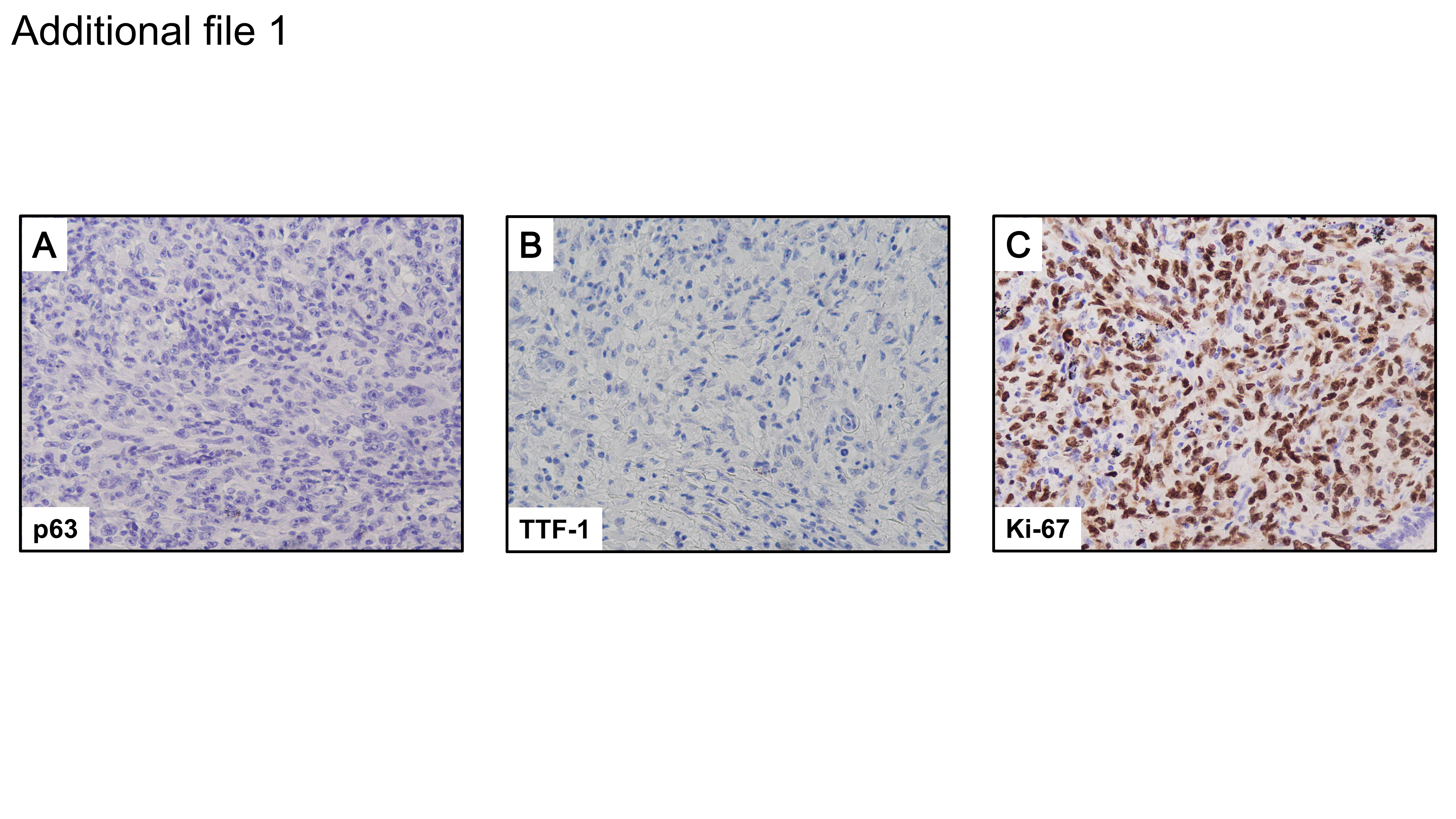

Supplement: Supplementary file 1 — (A, B) Immunohistochemistry showed that the lung tumor cells were negative for p63 (A; × 200) and thyroid transcription factor-1 (B; × 200). (C) The Ki-67 cell proliferation index was approximately 70–80% (× 200). (TIF 9365 kb) [file 12957_2019_1671_MOESM1_ESM.tif]
